# Multimodal Integration in Health Care: Development With Applications in Disease Management

**DOI:** 10.2196/76557

**Published:** 2025-08-21

**Authors:** Yan Hao, Chao Cheng, Juanjuan Li, Hongwen Li, Xingsi Di, Xiaoxia Zeng, Shoumei Jin, Xiaodong Han, Chongsong Liu, Qianqian Wang, Bingying Luo, Xianhai Zeng, Ke Li

**Affiliations:** 1 Department of Otolaryngology, Shenzhen Longgang Otolaryngology Hospital & Shenzhen Otolaryngology Research Institute, 186 Huangge Road, Longcheng Subdistrict, Longgang District, Shenzhen, Guangdong, 518172, China, 86 (755)28989999; 2 Department of Dentistry, Shenzhen Longgang Otolaryngology Hospital & Shenzhen Otolaryngology Research Institute, Shenzhen, Guangdong, China; 3 School of Law, Guangzhou University, Guangzhou, Guangdong, China; 4 Department of Ophthalmology, Shenzhen Longgang Otolaryngology Hospital & Shenzhen Otolaryngology Research Institute, Shenzhen, Guangdong, China; 5 Department of Medical Imaging, Shenzhen Longgang Otolaryngology Hospital & Shenzhen Otolaryngology Research Institute, Shenzhen, Guangdong, China; 6 Department of Immunology, Tianjin Medical University, Tianjin, China

**Keywords:** multimodal integration, healthcare, personalized medicine, artificial intelligence, digital health

## Abstract

Multimodal data integration has emerged as a transformative approach in the health care sector, systematically combining complementary biological and clinical data sources such as genomics, medical imaging, electronic health records, and wearable device outputs. This approach provides a multidimensional perspective of patient health that enhances the diagnosis, treatment, and management of various medical conditions. This viewpoint presents an overview of the current state of multimodal integration in health care, spanning clinical applications, current challenges, and future directions. We focus primarily on its applications across different disease domains, particularly in oncology and ophthalmology. Other diseases are briefly discussed due to the few available literature. In oncology, the integration of multimodal data enables more precise tumor characterization and personalized treatment plans. Multimodal fusion demonstrates accurate prediction of anti–human epidermal growth factor receptor 2 therapy response (area under the curve=0.91). In ophthalmology, multimodal integration through the combination of genetic and imaging data facilitates the early diagnosis of retinal diseases. However, substantial challenges remain regarding data standardization, model deployment, and model interpretability. We also highlight the future directions of multimodal integration, including its expanded disease applications, such as neurological and otolaryngological diseases, and the trend toward large-scale multimodal models, which enhance accuracy. Overall, the innovative potential of multimodal integration is expected to further revolutionize the health care industry, providing more comprehensive and personalized solutions for disease management.

## Introduction

In the realm of computer science, the concept of multimodal data refers to the integration and analysis of information from multiple sources or modalities. These modalities can include text, images, audio, video, and sensor data, among others [1]. The primary objective of multimodal data integration is to leverage the complementary strengths of different data types to gain a more comprehensive understanding of a given problem or phenomenon. By combining diverse data sources, multimodal approaches can enhance the accuracy, robustness, and depth of analysis [2,3].

In the context of health care, the application of multimodal data integration becomes even more critical due to the diversity of medical information. The health care sector generates vast amounts of data from a wide array of sources, including medical imaging (such as magnetic resonance imaging [MRI], computed tomography [CT] scans, and x-rays), laboratory test results, electronic health records (EHRs), wearable devices, and environmental sensors [4]. Medical imaging modalities provide detailed anatomical and functional views of the body. EHRs contain a wealth of clinical information, including patient history, diagnoses, treatments, and outcomes, which are essential for longitudinal health monitoring. Wearable devices continuously monitor physiological parameters, such as heart rate, blood pressure, and physical activity, providing real-time data on a patient’s health status. Each of these data types provides unique and valuable insights into patient health, but when considered in isolation, they may offer an incomplete or fragmented view. The integration of these diverse data sources enables a more nuanced and comprehensive understanding of patient health.

However, the integration and analysis of multimodal data in health care present significant difficulties. The sheer volume and heterogeneity of the data require sophisticated methodologies capable of handling large, complex datasets. This is where artificial intelligence (AI) and machine learning come into play. The development of multimodal AI is a rapidly evolving field. This approach has already shown promise in various areas of health care [5-7]. Through AI-driven integration of multimodal data, health care providers can achieve a more comprehensive understanding of patient conditions, leading to more accurate diagnoses, personalized treatments, and improved patient outcomes [8].

The future of multimodal integration in health care is promising, with ongoing research and technological advancements poised to further enhance its capabilities and applications. Emerging technologies, such as advanced imaging modalities, next-generation sequencing, and novel wearable devices, are expected to provide even richer datasets for integration [9]. In addition, the development of more sophisticated AI algorithms and data fusion techniques will enhance the ability to analyze and interpret complex multimodal data.

Despite the vast potential of multimodal integration in health care, several challenges remain to be addressed. First, data standardization and privacy protection require robust solutions while ensuring regulatory compliance. Second, model training and deployment face computational bottlenecks when processing large-scale and biased multimodal datasets. Third, model interpretability must be enhanced to provide clinically meaningful explanations that gain physician trust. Overcoming these barriers is critical for realizing the full clinical potential of multimodal health care systems.

The purpose of this viewpoint is to provide an overview of the current state of multimodal integration in health care, summarize its applications across key disease domains, and discuss the challenges and future directions in this rapidly evolving field. By examining the development and applications of multimodal integration across different disease domains, this viewpoint aims to offer insights into how this approach can further revolutionize the health care industry by providing more comprehensive and personalized solutions for disease management. The content of this study was informed by a systematic search of relevant studies (Multimedia Appendix 1).

## Applications

### Overview

This section focuses on 2 clinical domains that have seen particularly robust development of multimodal AI applications—oncology and ophthalmology. These specialties were selected due to their substantial body of published research and complex diagnostic requirements benefiting from multimodal data. As summarized in Table 1, we provide a summary of current multimodal developments in these fields.

**Table 1.** Multimodal artificial intelligence applications across specialties.

Disease and application directions	Specific examples
Oncology	
Enhanced tumor characterization	Tumor subtype and tumor microenvironment
Personalized treatment planning	Personalized radiotherapy and immunotherapy
Early detection and diagnosis	Early cancer detection
Predicting disease prognosis	Overall survival and progression-free survival
Ophthalmology	
Early diagnosis and risk stratification	Glaucoma and age-related macular degeneration
Ophthalmology imaging as a noninvasive predictive tool for circulatory system disease	Cardiovascular disease

### Application of Multimodal Data in Oncology

#### Overview

The integration of multimodal data in cancer care represents one of the most promising advancements in modern oncology. For example, advancements in quantitative multimodal imaging technologies involve the combination of multiple quantitative functional measurements, thereby providing a more comprehensive characterization of tumor phenotypes [10]. In addition, integrated genomic analysis methods can reveal dysregulation in biological functions and molecular pathways, offering new opportunities for personalized treatment and monitoring [11]. By combining diverse data sources, health care providers can achieve a more comprehensive understanding of cancer biology, leading to more accurate predictions of patient outcomes. This section explores the various applications of multimodal data in cancer care, highlighting specific case studies and the transformative impact of this approach.

#### Enhanced Tumor Characterization

One of the primary objectives of integrating multimodal data in cancer care is to achieve enhanced tumor characterization. Tumor characterization involves understanding the genetic, molecular, and phenotypic features of a tumor [12-14], which is essential for elucidating the nature and properties of the malignancy.

A key aspect of this process is the differentiation of tumor subtypes. Tumor subtypes refer to the classification of tumors into distinct categories. Differentiating tumor subtypes is essential because it allows for more precise diagnosis, prognosis, and the development of tailored treatment strategies, specific to the characteristics of each subtype [15]. Previous cancer subtypes were often classified based on gene expression profiles, such as the PAM50 method [16,17]. However, patients within the same group may still experience different outcomes [18], indicating the need for more accurate subtype classification methods. Pathological images and omics data are commonly used for accurate tumor classification through multimodal integration. The features derived from the fusion of image modality data with genomic and other omics data can predict breast cancer subtypes [19]. Typically, dedicated feature extractors are used for each modality. A trained convolutional neural network model captures deep features from pathological images, while a trained deep neural network model extracts features from genomic and other omics data. These multimodal features are then integrated through a fusion model to achieve an accurate prediction of breast cancer molecular subtypes. This integrative approach can also be extended to other tumor types and even pan-cancer studies to support the prediction of cancer subtypes and severity [20-22]. A large-scale study integrated transcriptome, exome, and pathology data from over 200,000 tumors to develop a multilineage cancer subtype classifier [18].

The tumor microenvironment (TME) plays a crucial role in tumor initiation, progression, metastasis, and resistance to therapy [23,24]. In recent years, advancements in new technologies such as single-cell and spatial technologies [25] have provided fine-grained resolution of TME, significantly enhancing our understanding of cellular interactions at both single-cell and spatial dimensions [26,27]. Besides, the use of multimodal nanosensors can achieve real-time monitoring within the TME [28]. Using multimodal features extracted from single-cell and spatial transcriptomics reveals immunotherapy-relevant non–squamous cell carcinoma (non–small cell lung cancer [NSCLC]) TME heterogeneity [29]. The combination of the 2 modalities and multiplexed ion beam imaging identifies distinct tumor subgroups and a cancer-specific tumor-specific keratinocyte [30]. Spatial multiomics delineate core and margin compartments in oral squamous cell carcinoma, with metabolically active margins demonstrating elevated adenosine triphosphate production to fuel invasion [31]. In cross-modal applications, gene expression can be predicted from histopathological images of breast cancer tissue with a resolution of 100 µm [32]. Conversely, spatial transcriptomic features can better characterize breast cancer tissue sections, revealing hidden histological features [33]. By extracting interpretable features from pathological slides, it is also possible to predict different molecular phenotypes [34]. These methods provide a comprehensive, quantitative, and interpretable window into the composition and spatial structure of the TME.

#### Personalized Treatment Planning

Another critical objective of multimodal data integration in cancer care is personalized treatment planning. Personalized treatment involves tailoring medical interventions to the individual characteristics of each patient, taking into account their tumor biology and overall health status. By integrating data from multiple sources, health care providers can develop more precise and personalized treatment plans that improve patient outcomes.

In terms of radiation therapy, using multimodal scanning techniques and mathematical models, it is possible to design personalized radiotherapy plans for glioblastoma patients. By integrating high-resolution MRI scans and metabolic profiles, this approach enables more accurate inference of tumor cell density, thereby optimizing radiotherapy regimens and reducing damage to healthy tissue [35]. The integration of biological information-driven multimodal imaging techniques allows physicians to better understand the spatial and temporal heterogeneity of tumors to develop personalized radiotherapy regimens [36].

In the trend of precision medicine, another therapeutic approach is immunotherapy. Immune checkpoint blockade can unleash immune cells to reinvigorate antitumor immunity [37]. Multiple phase III clinical trials have demonstrated that the anti–programmed cell death protein 1 antibody nivolumab significantly improves overall survival with a favorable safety profile in patients with NSCLC [38]. Although single-modality biomarkers can predict responses to immune checkpoint blockade, their predictive power is not always satisfactory. Activating an antitumor immune response through immunotherapy involves a series of complex events that require the interaction of multiple cell types [39]. Therefore, achieving precision immunotherapy necessitates integrating multiple data modalities and adopting a holistic approach to analyze the human TME. Translating these multimodal factors into clinically usable predictive markers facilitates the selection of optimal immunotherapy. Combining the informational content present in routine diagnostic data, including annotated CT scans, digitized immunohistochemistry slides, and common genomic alterations in NSCLC, can improve the prediction of responses to programmed cell death protein 1 or programmed cell death-ligand 1 blockade [40]. Multi-modal model by Chen et al [41] can predict the response to anti–human epidermal growth factor receptor 2 combined immunotherapy using multimodal radiology, pathology, and clinical information, achieving an area under the curve (AUC) of 0.91. Furthermore, the application of multimodal approaches in targeted cancer therapy has demonstrated significant potential. Integrating radiomic phenotypes with liquid biopsy data can enhance the predictive accuracy for the efficacy of epidermal growth factor receptor inhibitors [42].

#### Early Detection and Diagnosis

Early detection and diagnosis of cancer are crucial for improving patient outcomes, as early-stage cancers are often more treatable and have better prognoses [43]. Multimodal data integration plays a vital role in enhancing the accuracy and timeliness of cancer detection and diagnosis.

Liquid biopsy is a noninvasive technique that involves the collection of nonsolid samples, providing possibilities for early cancer detection and longitudinal tracking [44]. This technology includes circulating tumor cells shed from primary and metastatic tumors, as well as circulating tumor DNA (ctDNA) [45]. ctDNA can detect trace amounts of tumor DNA even before the tumor manifests obvious symptoms or becomes visible through imaging. Numerous studies and articles have used ctDNA in combination with various other modalities for early cancer prediction, including lung cancer [46], breast cancer [47], and colorectal cancer [48]. Cell-free DNA is a substance that is consistently present in plasma and has been receiving increasing attention. Combining cell-free DNA with other modalities can be used for highly specific early detection across multiple cancer types [49-51]. AutoCancer uses a transformer model to integrate multiple modalities, including liquid biopsy, mutation, and clinical data, achieving accurate early cancer detection in both lung cancer and pan-cancer analyses [52]. Multimodal models that integrate genomic features and clinical data have also demonstrated excellent performance in the early detection of colorectal cancer, with an AUC of 0.98 in the validation set and a sensitivity and specificity of more than 90% [49].

#### Predicting Disease Prognosis

Prognosis involves assessing the risk of future outcomes based on an individual’s clinical and nonclinical characteristics. These outcomes are typically specific events, such as death or complications, but they can also be quantitative measures, such as disease progression, changes in pain levels, or quality of life [53]. Predicting disease prognosis is a critical aspect of cancer care, as it allows for timely interventions and improved long-term outcomes. Multimodal data integration enhances the ability to predict disease prognosis.

Prognosis in tumor research can be divided into 2 key areas: recurrence and survival. In the context of recurrence, a retrospective analysis and multicenter validation study involving over 2000 patients demonstrated that a multimodal recurrence score, which integrated clinical, genomic, and histopathological data, accurately predicted postoperative local recurrence of renal cell carcinoma [54]. Combining the emerging tool of habitat imaging with traditional gene expression and clinical data enables noninvasive stratification of patients with NSCLC, enhancing the prediction of recurrence risk [55]. In another study, algorithms were developed based on structured clinical and administrative data to detect recurrence in lung and colorectal cancer patients. By using EHRs and tumor registry data, these algorithms successfully improved the accuracy of recurrence detection [56].

Regarding survival, an increasing number of studies have adopted multimodal approaches to predict patient survival [57-61]. By integrating data from various sources, these studies have achieved accurate survival predictions across multiple tumor types, including overall survival, 5-year survival rates, and progression-free survival.

### Application of Multimodal Data in Ophthalmology

#### Overview

Ophthalmology, the medical specialty focused on the diagnosis and treatment of eye disorders, has experienced significant advancements through the integration of multimodal data. Advanced imaging techniques are central to ophthalmology, providing detailed visualizations of the retina, optic nerve, and other ocular structures [62]. Optical coherence tomography (OCT) is a widely used imaging modality that offers high-resolution cross-sectional images of the retina, enabling the detection of structural abnormalities and disease progression. Fundus photography and fluorescein angiography provide additional insights into the retinal vasculature and blood flow, which are critical for diagnosing and managing conditions like diabetic retinopathy and retinal vein occlusion. These imaging techniques, when integrated, offer a comprehensive view of both the structural and genetic factors contributing to ocular diseases. The fusion of these data types enables early diagnosis, personalized treatment plans, and continuous monitoring of disease progression and response to therapy, particularly in conditions like age-related macular degeneration (AMD), diabetic retinopathy, and glaucoma [63].

#### Early Diagnosis and Risk Stratification

The integration of these diverse data types in ophthalmology achieves several important objectives. Early diagnosis and risk stratification are critical for managing ocular diseases, and the combination of genetic, imaging, and clinical data enables the identification of early signs of eye conditions and stratification of patients based on their risk profiles.

Color fundus photography and OCT are 2 of the most cost-effective tools for glaucoma screening. Mehta et al [64] developed a high-performance multimodal glaucoma detection system by integrating OCT volumes, fundus photographs, and clinical data. Their approach combined features extracted from individual modalities, followed by gradient boosting decision trees for final multimodal construction. The model was rigorously developed and validated on a cohort of 96,020 UK Biobank participants, demonstrating both excellent discriminative performance (AUC=0.97). Importantly, the architecture maintained clinical interpretability through comprehensive feature importance analysis [64]. Other multimodal models for glaucoma and its grading detection, based on modalities, such as OCT and fundus images, have also achieved AUC exceeding 0.90 [65-67]. By using a dual-stream convolutional neural network model to extract features from OCT and color fundus photographs, AMD can be classified into 3 categories—normal fundus, dry AMD, and wet AMD [68]. Another study enrolled 75 participants from optometry clinics in Auckland and Milford Eye Clinic, New Zealand. By stratifying subjects into young healthy controls, older adult healthy controls, and moderate dry AMD groups, the multimodal diagnostic system achieved 96% classification accuracy [69]. In addition, the use of multimodal data can also identify polypoidal choroidal vasculopathy [70], dry eye disease [71], and diabetic retinopathy [72-75]. There is also comprehensive work demonstrating that multimodal deep learning (DL) models, which use combined color fundus photography and OCT image sequences as input, can be used to simultaneously detect multiple common retinal diseases [76,77].

#### Ophthalmology Imaging as a Noninvasive Predictive Tool for Circulatory System Disease

Currently, the diagnosis and treatment of circulatory system disease primarily rely on imaging examinations such as MRI, coronary CT angiography, and coronary angiography. These examinations are not only expensive and time-consuming but also partially invasive and require a high level of professional expertise from the operators. Consequently, early screening and long-term follow-up examinations are challenging to implement in regions with limited medical resources. To better achieve early warning and assessment of circulatory system disease, there is a continuous need to develop new diagnostic tools that are noninvasive, convenient, and efficient.

The microcirculation of the retina is part of the body’s microcirculation system and shares similar embryological origins and pathophysiological characteristics with the cardiovascular system [78]. Numerous studies have identified retinal imaging biomarkers associated with early cardiovascular diseases (CVDs) lesions and prognosis, demonstrating the significant value of retinal imaging in CVD screening and prognostic evaluation [79,80].

Al-Absi et al [81] used a multimodal approach integrating retinal images and dual-energy x-ray absorptiometry data to diagnose CVD in a Qatari cohort. The multimodal model achieved 78.3% accuracy, outperforming unimodal models [81]. Notably, their model is interpretable, using Gradient-weighted Class Activation Mapping (Grad-CAM) to highlight the areas of interest in retinal images that most influenced the decisions of the proposed DL model. A study using clinical information and fundus photographs from the UK Biobank demonstrated a significant association between the incidence of CVD in high-risk patients and multimodal predicted risk (hazard ratio 6.28, 95% CI 4.72‐8.34), and visualized feature importance [82].

## Challenges in Multimodal Health Care

While the integration of multimodal data in health care holds great promise, it also presents several significant challenges that need to be addressed.

### Data Standardization and Privacy

One of the primary challenges in multimodal health care is integrating diverse medical data sources with varying formats, resolutions, and quality levels [83]. Inconsistent data collection practices, missing entries, and recording errors can compromise model reliability, necessitating robust standardization protocols [84]. Effective multimodal integration requires comprehensive data cleaning, validation, and preprocessing to create cohesive, high-quality datasets that support accurate predictive analytics. The growing availability of novel health care data sources presents both opportunities for personalized medicine and challenges for systematic integration.

The use of multimodal data in health care raises significant concerns about data privacy and security. Medical data is highly sensitive, and ensuring its protection is paramount. Regulatory frameworks, such as the Health Insurance Portability and Accountability Act in the United States and the General Data Protection Regulation in the European Union, are essential for protecting patient privacy and ensuring data security. But the concept of health information privacy continues to evolve over time. As new technologies and data sources emerge, it is essential to update and adapt these legal frameworks to reflect new realities [85]. The use of multimodal data raises significant privacy concerns [86]. Implementing robust data encryption, secure data storage, and strict access controls are essential measures to protect patient information [87]. Comprehensive data governance frameworks must establish clear guidelines for responsible and transparent multimodal data usage, while carefully balancing potential risks and benefits for participants, researchers, and society at large [88]. Effective implementation requires developing robust data sharing agreements, establishing independent oversight committees, and maintaining ongoing engagement with research participants and other stakeholders [89]. In addition, developing secure data sharing protocols and anonymization techniques can help mitigate risks while enabling the effective use of multimodal data for research and clinical applications [90]. Ensuring data privacy and security is fundamental to maintaining patient trust and the ethical use of medical data.

The initial phase of multimodal health care requires systematic collection of standardized data following heterogeneity resolution, coupled with privacy protection through secure protocols. Integrating rigorous data processing with ethically compliant governance frameworks enables usage of diverse datasets for precision medicine while safeguarding sensitive information. This equilibrium is critical for advancing research ethically and maintaining public trust in medical AI applications.

### Model Training and Deployment

Multimodal models demand substantial computational resources for both training and inference. The complexity of these models often results in extended training times and significant costs, which can be prohibitive for many health care institutions [91,92]. Training these models requires high-performance computing environments equipped with powerful Graphics Processing Units or Tensor Processing Units, which are not always accessible to all institutions. Furthermore, the inference phase, where the trained model is applied to new data, can also be resource-intensive, particularly when dealing with large-scale datasets or real-time applications [93]. This computational burden can limit the scalability and practical deployment of multimodal models in clinical settings.

Beyond computational constraints, biases in training data pose a significant challenge to multimodal fusion. Biases may arise from uneven data distribution, inconsistent annotation quality, or systemic disparities in data collection. AI-driven decisions are fundamentally shaped by their initial training data. If the underlying datasets contain biases or inequities, the resulting algorithms risk perpetuating prejudice, incomplete representations, or discriminatory outcomes—potentially amplifying systemic inequalities [94]. To counteract these biases, strategies such as bias-aware sampling and fairness constraints during model optimization can be implemented. While some AI developers claim their algorithmic systems can mitigate biases, critics maintain that algorithms alone cannot eradicate discrimination, as they may inadvertently perpetuate existing bias in training data [95]. This tension highlights the need for complementary strategies (ie, rigorous dataset curation to ensure diversity and continuous monitoring for disparate impacts) [96].

Training and running multimodal models demand expensive hardware, limiting clinical adoption. Meanwhile, biased training data can perpetuate health care disparities. While optimization techniques and bias mitigation strategies help, robust data curation and ongoing monitoring of potentially biased data remain essential for practical, equitable deployment.

### Model Interpretability

While multimodal models can achieve high accuracy, their complexity often makes them difficult to interpret. This lack of interpretability poses a significant barrier to their adoption in clinical practice, as clinicians and patients need to understand the rationale behind model predictions to trust and effectively use these tools [97]. Enhancing the interpretability and transparency of multimodal models is therefore crucial [98]. Techniques, such as explainable artificial intelligence (XAI), can play a pivotal role in this regard [99]. XAI methods aim to make the decision-making processes of AI models more understandable to humans by providing explanations that are both accurate and comprehensible. Classical XAI approaches include attention mechanisms and Grad-CAM. Attention scores highlight relevant regions through forward propagation, while Grad-CAM reveals feature significance by capturing gradient changes during backpropagation [100].

Attention mechanisms were originally developed to help neural networks focus on the most relevant parts of input data when making predictions. The core principle involves calculating attention weights—numerical scores that determine how much each input element (eg, words in text or regions in an image) should influence the model’s output [101]. MedFuseNet [102] uses an image attention mechanism to dynamically focus on the most clinically relevant regions of medical images corresponding to the input textual queries. Visualization of the attention matrices reveals that the model consistently attends to anatomically discriminative regions of target organs, demonstrating its capability to identify pathologically significant features. StereoMM [103] enables quantitative analysis of cross-attention matrices to determine the relative contribution weights of different modalities during fusion, thereby offering interpretable insights into the prioritization of modalities by the model in its decision-making process. Nevertheless, attention weights primarily reflect statistical correlations rather than causal relationships. The fact that a feature receives high attention does not necessarily imply it was determinative for the model’s prediction. Compounding this issue, empirical studies have demonstrated that substantially different attention weight distributions can yield identical model outputs [104]. These limitations raise questions about the validity of using attention mechanisms as reliable tools for explaining neural network behavior, making an ongoing subject of debate in the machine learning community [105].

Grad-CAM generates explanations by computing gradients from the final convolutional layer, highlighting prediction-relevant regions [106]. This interpretability method helps detect invalid decision patterns. For instance, if highest activations appear on imaging artifacts rather than anatomical structures, it exposes critical model flaws. In a clinical study using brain MRI for classification of multiple sclerosis subtypes, Grad-CAM–generated heatmaps consistently and distinctly highlighted brain regions critical for differentiating between subtypes, thereby demonstrating the validity and explanatory power. Furthermore, Grad-CAM analysis identified previously unrecognized neuroanatomical loci, offering novel insights into disease progression mechanisms and potentially revealing new imaging biomarkers or therapeutic targets [107]. It should be noted that Grad-CAM offers qualitative visualization of model decisions, not quantitative validation. Its clinical relevance must be determined through physician assessment of the identified features [108].

Multimodal AI models face a key challenge—balancing high accuracy with clinical interpretability. Current XAI methods offer partial solutions, but with important limitations. Both methods produce explanations that require clinical validation, and physician expertise remains essential to assess biological plausibility. These limitations highlight the need for XAI approaches that provide both technical transparency and clinically meaningful explanations to enable trustworthy AI adoption in health care.

## The Development Direction of Multimodal Technology: Expanding Disease Applications

The development of multimodal technology encompasses broader applications across various diseases and the advancement of large-scale models. With technological progress, multimodal approaches are no longer limited to the diagnosis and prognosis of cancer and ophthalmic diseases but are expanding into CVD, neurological disorders, metabolic diseases, otolaryngology, and more.

In the field of CVD, multimodal technology can combine data from cardiac MRI, coronary CT, echocardiography, and biomarkers to provide a more comprehensive assessment of heart health [87]. For example, integrating these data can more accurately predict the risk of ischemic heart disease [109,110], coronary artery disease [111], assess cardiac function [112], and detect disease subgroups plans [113]. In addition, multimodal technology can be used to monitor the treatment effects and disease progression in heart disease patients, allowing timely adjustments to treatment strategies and improving patient survival rates and quality of life [114].

In the realm of neurological disorders, multimodal technology also holds significant promise. A proposed model demonstrates robust multimodal integration capabilities, effectively combining both imaging and nonimaging clinical data to achieve accurate differential diagnosis of Alzheimer disease, with discriminative performance exceeding AUC values of 0.9 across multiple diagnostic tasks [115]. By combining brain MRI, functional MRI, electroencephalography, and genomic data, researchers can gain a more comprehensive understanding of the pathophysiology of diseases such as Alzheimer [116], Parkinson [117], and multiple sclerosis [118]. Integrating these data can aid in the early diagnosis of these diseases and assess disease severity.

In the field of metabolic diseases, multimodal technology also has important applications. Integrating clinical documentation with structured laboratory data significantly improves the predictive performance of unimodal machine learning models for early-stage type 2 diabetes mellitus detection. The model achieved an AUC greater than 0.70 for new-onset type 2 diabetes mellitus prediction [119]. By integrating metabolomics, genomics, imaging, and clinical data, researchers can gain a more comprehensive understanding of the pathophysiology of diseases, such as obesity [120] and fatty liver disease [121]. Integrating these data can aid in the early diagnosis of these diseases and assess disease status.

In the field of otolaryngology, the automatic classification of parotid gland tumors based on multimodal MRI sequences shows promise for improving diagnostic decision-making in clinical settings [122]. The integration of CT and MRI enables precise tumor segmentation of oropharyngeal squamous cell carcinoma, resulting in higher dice similarity coefficients and lower Hausdorff distances [123]. Combining otoscopic images and wideband tympanometry enables the automatic detection of otitis media [124]. Institutions have recognized the importance of collecting multimodal data for interdisciplinary audiology research and have developed a multimodal database that can be used for algorithm development [125].

## The Trend Toward Large Language Models

Large language models (LLMs) are foundational pretrained AI systems capable of processing and generating human-like text [126]. Their key advantage lies in capturing complex semantic relationships within language data. Building upon LLMs, large multimodal models extend these capabilities to integrate and analyze diverse data types (text, images, genomic data, etc), achieving significant advancements and breakthroughs, gradually forming the rudiments of artificial general intelligence [127]. The trend toward LLM in multimodal technology enhances the accuracy and robustness of disease prediction and diagnosis by capturing complex relationships between different data types [128,129].

For example, transformer models, which have achieved remarkable success in natural language processing and computer vision, are now being applied to the integration and analysis of multimodal data [130]. The transformer-based unified multimodal diagnostic transformer model is capable of directly generating diagnostic results for lung diseases from multimodal input data [131].

Furthermore, LLMs have stronger generalization capabilities, allowing them to be applied across various diseases and populations. This general-purpose approach not only enhances diagnostic accuracy but also reduces the cost and complexity of training and deploying multiple specialized models. For instance, a single large multimodal model could be used for the diagnosis and prognosis of cancer, aging and age-related diseases [132], CVDs, neurological disorders, and metabolic diseases, streamlining the process and improving efficiency.

Another important aspect of LLMs is their interpretability, primarily achieved through the use of attention weights. Although DL models are often considered “black boxes,” recent advancements have focused on improving model transparency. Attention mechanisms enhance interpretability by identifying and emphasizing the most critical features in the input data, allowing attention to be visualized as regions of information that contribute to decision-making [133,134]. By visualizing the distribution of attention weights, one can extract the content with high attention weights, which often have a greater impact on the final outcome prediction [135].

In summary, the trend toward LLMs in multimodal development is poised to bring significant innovations and breakthroughs to the medical field. By leveraging the power of large-scale, multimodal datasets and advanced neural network architectures, researchers can achieve more accurate and comprehensive disease predictions and diagnoses.

## Supplementary material

10.2196/76557 Multimedia Appendix 1 Additional material.

## Abbreviations

AI: artificial intelligence
AMD: age-related macular degeneration
AUC: area under the curve
CT: computed tomography
ctDNA: circulating tumor DNA
CVD: cardiovascular disease
DL: deep learning
EHR: electronic health record
Grad-CAM: Gradient-weighted Class Activation Mapping
LLM: large language model
MRI: magnetic resonance imaging
NSCLC: non–small cell lung cancer
OCT: optical coherence tomography
TME: tumor microenvironment
XAI: explainable artificial intelligence
