# Supplementary material for: Multimodal Integration in Health Care: Development With Applications in Disease Management
Source: J Med Internet Res. 2025 Aug 21;27:e76557. doi: 10.2196/76557 (PMC12370271; doi:10.2196/76557)
Supplement: Multimedia Appendix 1 [file jmir-v27-e76557-s001.docx]

Supplementary Materials for

**Multimodal Integration in Healthcare: Development with Applications in Disease Management**

**This PDF file includes:**

Supplementary Text (Methods)

## Methods

To ensure a comprehensive and transparent viewpoint of multimodal integration in healthcare, a systematic search strategy was employed to identify relevant studies. The following databases were searched: PubMed, Web of Science, IEEE Xplore, and arXiv. The search was conducted from January 2009 to January 2025 to capture the most recent advancements and developments in the field.

**Search Terms**

The following key terms and their combinations were used to identify relevant articles:

- "multimodal integration" AND "healthcare"
- "multimodal data" AND "medicine"
- "multimodal AI" AND "disease management"
- "multimodal imaging" AND "genomics"
- "multimodal models" AND "clinical applications"

**Inclusion and Exclusion Criteria**

**Inclusion Criteria:**

1. Studies that focused on the integration of multimodal data in healthcare.
2. Articles that provided original research data or comprehensive reviews on the application of multimodal integration in disease management.
3. Articles that discussed the use of multimodal integration in oncology, ophthalmology, cardiovascular diseases, neurological disorders, metabolic diseases, and otolaryngology.

**Exclusion Criteria:**

1. Studies with insufficient data or those that did not focus on multimodal integration.
2. Articles unrelated to the main theme of multimodal integration in disease management.

**Screening Flow**

The screening process followed these steps:

1. Initial Search: A total of 495 articles were retrieved from the databases using the specified search terms.
2. Title and Abstract Screening: Titles and abstracts were screened for relevance to the topic of multimodal integration in healthcare. This step excluded 208 articles that did not meet the inclusion criteria.
3. Full-Text Evaluation: The remaining 163 articles were evaluated in full text. Articles that did not provide sufficient data or did not focus on multimodal integration were excluded, resulting in a final selection of 135 articles.
